# Differential Impacts of Water Table and Temperature on Bacterial Communities in Pore Water From a Subalpine Peatland, Central China

**DOI:** 10.3389/fmicb.2021.649981

**Published:** 2021-05-28

**Authors:** Wen Tian, Xing Xiang, Hongmei Wang

**Affiliations:** ^1^State Key Laboratory of Biogeology and Environmental Geology, China University of Geosciences, Wuhan, China; ^2^School of Environmental Studies, China University of Geosciences, Wuhan, China; ^3^College of Life Science, Shangrao Normal University, Shangrao, China

**Keywords:** bacterial structure, metabolic diversity, bacterial phenotype, co-occurrence network, water table, temperature

## Abstract

The level of water table and temperature are two environmental variables shaping soil bacterial communities, particularly in peatland ecosystems. However, discerning the specific impact of these two factors on bacterial communities in natural ecosystems is challenging. To address this issue, we collected pore water samples across different months (August and November in 2017 and May 2018) with a gradient of water table changes and temperatures at the Dajiuhu peatland, Central China. The samples were analyzed with 16S rRNA high-throughput sequencing and Biolog EcoMicroplates. Bacterial communities varied in the relative abundances of dominant taxa and harbored exclusive indicator operational taxonomic units across the different months. Despite these differences, bacterial communities showed high similarities in carbon utilization, with preferences for esters (pyruvic acid methyl ester, Tween 40, Tween 80, and D-galactonic acid γ-lactone), amino acids (L-arginine and L-threonine), and amines (phenylethylamine and putrescine). However, rates of carbon utilization (as indicated by average well-color development) and metabolic diversity (McIntosh and Shannon index) in May and August were higher than those in November. Redundancy analysis revealed that the seasonal variations in bacterial communities were significantly impacted by the level of the water table, whereas the temperature had a fundamental role in bacterial carbon utilization rate. Co-occurrence analysis identified *Sphingomonas*, *Mucilaginibacter*, *Novosphingobium*, *Lacunisphaera*, *Herminiimonas*, and *Bradyrhizobium* as keystone species, which may involve in the utilization of organic compounds such as amino acids, phenols, and others. Our findings suggest that bacterial community functions were more stable than their compositions in the context of water table changes. These findings significantly expand our current understanding of the variations of bacterial community structures and metabolic functions in peatland ecosystems in the context of global warming and fluctuation of the water table.

## Introduction

Peatlands are vast reservoirs of organic carbon, which store approximately 33% of the terrestrial carbon (Yu et al., 2010), and thus play an important role in the global carbon budget. Microbial communities in peatland ecosystems are key players in nutrient cycles, carbon sequestration, and greenhouse gas emissions (Kip et al., 2010; Dedysh, 2011; Yang et al., 2017). Microbial communities in peatlands are sensitive to water table (WT) fluctuations (Jaatinen et al., 2007; Urbanová and Bárta, 2016; Tian et al., 2019) and temperature changes (Fenner et al., 2007; Kolton et al., 2019), which are two major factors of concern in the context of climate change. Understanding the variations of microbial community structures and carbon utilization with WT fluctuations and temperature changes will help us comprehend the dynamics of carbon cycle in peatland ecosystems under climate change scenarios.

Water table fluctuations have been demonstrated to impact microbial diversity and activities. The level of the WT can affect microbial biomass (Mäkiranta et al., 2009) and greenhouse gas emissions (Moore and Dalva, 1993; Strack et al., 2004; Hoyos-Santillan et al., 2019) in peatlands. Short-term drought will increase the bacterial diversity and microbial degradation of phenolic compounds (Kwon et al., 2013). However, long-term drainage could result in the decrease of microbial diversity and activity (Urbanová and Bárta, 2016). Storm water or flooding can cause the loss of microbial diversity and increase the dissolved organic carbon (DOC) in pore water (Fenner et al., 2005), favoring for methanogens (Freitag et al., 2010; Järveoja et al., 2016). In contrast, the increase of the oxic layer promotes aerobic respiration and diminishes substrates for anaerobic degradation and further depresses the activity and diversity of methanogens (Urbanová et al., 2011; Yrjälä et al., 2011).

Besides WT, temperature also influences microbial community structure (Guo et al., 2018) and carbon utilization (Wang R. et al., 2018). Previous studies have demonstrated temperature effects on the quality and quantity of soil organic matter (Fissore et al., 2008), microbial metabolic rates and pathways (Davidson and Janssens, 2006; Dijkstra et al., 2011), and microbial community composition and physiology (Garcia-Pichel et al., 2013). Elevated temperature may increase species diversity, metabolic activity and population growth rate (Zhou et al., 2016), and bacterial biomass (Smoot and Findlay, 2001). It is known to stimulate the biodegradation of organic matter (Kallenbach et al., 2016) via enhancing enzyme activity (Alvarez et al., 2018) and substrate bioavailability (Qin et al., 2019).

In peatland ecosystems, pore water has a vital role in nutrient transport and as a pool of inorganic and organic compounds, and the geochemistry of peat pore water may impact peatland biota (Ulanowski and Branfireun, 2013). Vegetation and sediments are interdependent and connected by the physical, hydrological, biological, and chemical processes in pore water. For instance, fluxes of trace elements and nutrients from pore water to plants are crucial processes (Carling et al., 2013; Kloss et al., 2015). Sediments serve as important sources of trace element and nutrients to pore water via diffusion, dissolution, and bioturbation (Benoit et al., 2009). Previous studies mainly focused on hydrochemical properties and geochemical processes of pore water in peatlands (Ulanowski and Branfireun, 2013; Haapalehto et al., 2014; Juckers and Watmough, 2014). For example, the concentrations of dissolved organic carbon (DOC) in peatlands have increased over last decades due to warming, enhanced precipitation, and changes in atmospheric deposition (Freeman et al., 2001; Kane et al., 2014). DOC is closely linked with the decomposition rate of organic matter, which is strongly affected by changes in the WT (Sapek et al., 2009) and temperature (Jassey et al., 2012). WT and temperature are central, dynamic factors in physicochemical characteristics of peat pore water and accompany methane oxidation (Hornibrook et al., 2009; Putkinen et al., 2012). The responses of microbial communities to changes in peat pore water remain poorly understood. We hypothesize that WT level and temperature may have differential effects on bacterial communities and microbial degradation of organic matters in pore water.

One common approach used to assess the metabolic activities of heterotrophs, using organic carbon as the carbon source, is the community-level physiological profiling analysis via Biolog EcoMicroplates (Garland and Mills, 1991). Although Biolog technology is sensitive to inoculum density and cannot reflect the potential metabolic diversity *in situ* (Xue et al., 2008), it can detect metabolic activities of culturable microbes and relatively quickly characterize the ecological status of microbial communities (Fisk et al., 2003). This method has been successfully applied to characterize the metabolic activities of heterotrophs in various environmental samples such as sediments (Salomo et al., 2009), wastewater (Zhang et al., 2014), groundwater (Tiquia, 2010), soil (Wang et al., 2019), and compost (Zhou et al., 2019). The next-generation sequencing technology has greatly expanded our knowledge of microbial community composition via 16S rRNA gene sequencing from environmental samples (Thompson et al., 2017). The coupling of high-throughput sequencing and Biolog technologies should provide valuable information about the associations between microbial community structures and their ecological functions in peatland ecosystems.

To test our hypothesis, we combined the high-throughput sequencing technique and Biolog technology to characterize the diversity of bacterial communities in peatland pore water samples. The purpose was to discern between effects brought about by changes in the WT and temperature on bacterial communities and their carbon utilization at the Dajiuhu peatland across spring, summer, and fall. The results provide substantive information on the variation of bacterial communities and the impacts of WT and temperature changes on carbon cycling.

## Materials and Methods

### Sampling

Dajiuhu (1,730 m a.s.l., 31°28′ N, 110°0′ E), a subalpine peatland, is in Shennongjia at the middle reaches of Yangtze River, central China (Supplementary Figure 1), which has developed in the closed intermontane basin since the last deglaciation. The area of peatland is ca. 16 km^2^, and the mean peat thickness reaches >2 m (Huang et al., 2018). The local climate is dominated by the East Asian monsoon, with a mean annual rainfall of 1,560 mm and temperature of 7.2°C, respectively. Generally, the wet season is from April to September, and dry season is from October to March. The vegetation mainly consists of *Carex* spp., *Sphagnum palustre*, *Sanguisorba officinalis*, and *Euphorbia esula* accompanying shrubs (Tian et al., 2019). *Sphagnum* mosses are known as the major charcoal-forming plants in acidic peatlands and greatly contribute to peatland development (Tian et al., 2020). Twelve plots dominated by *S. palustre* were selected for sampling across three seasons to investigate the impact of WT on bacterial communities in the Dajiuhu peatland (Supplementary Figure 1).

To measure WT, 12 PVC pipes (1.5 m in length and 32 mm in diameter) were perforated with holes (6 mm in diameter) on the wall. The holes were separated with an interval of 20 cm along the entire pipe. The bottom of the perforated pipes was sealed to prevent ingress of sediment, and the top of pipe was covered with a sealed plastic bag. The pipes were drilled into the peat at each plot, and the level of the WT was determined with an Odyssey Logger (Dataflow Systems Ltd., Christchurch, New Zealand). In parallel, another set of 12 pipes was drilled to the same depth with the 12 pipes mentioned above but were perforated according to WT detected. Pore water samples from different WT depths were collected by a peristaltic pump with Teflon tube from the pipes in August and November 2017 and May 2018. Sampling in February was not practical because of the snowy and frozen season. Water samples (2 L in volume for each sampling plot) were immediately filtered through 0.22-μm sterile membranes to collect microbial cells in the field and aliquots of 50 mL water samples were kept in sterile centrifuge tubes at 4°C for the Biolog EcoMicroplate inoculation. The monthly mean temperatures were 18.2, 2.9, and 13.2°C, and monthly precipitations were 195, 63, and 363 mm in August, November 2017, and May 2018, respectively. All samples were transported to the geomicrobiology laboratory at China University of Geosciences within 12 h on ice.

### Hydrological and Physicochemical Analysis

Pore water temperature (PWT), dissolved oxygen (DO), pH, electrical conductivity (EC), and oxidation–reduction potential (ORP) were measured *in situ* using an HQ40d multiparameter meter (HACH, Loveland, CO, United States). DOC in the water samples was analyzed with an Aurora 1030W TOC Analyzer (OI Analytical, College Station, TX, United States) after filtration with 0.22-μm membrane.

### Biolog EcoMicroplates Inoculation

Aliquots of 100-μL peat pore water samples were added into the 96-well EcoMicroplates, which were preheated for 30 min at 25°C. The inoculated microplates were incubated at 25°C in the dark, and the absorbance of microplates was detected at 590 and 750 nm per 12 h for 240 h in total by a Biolog Microstation (Biolog, Hayward, CA, United States).

### DNA Extraction, Amplicon Sequencing, and Quantitative Polymerase Chain Reaction

The 0.22-μm membranes with microbial cells were used to extract total DNA with a PowerWater DNA Isolation Kit (QIAGEN, Düsseldorf). DNA concentration was detected with a NanoDrop 2000 spectrophotometer (Thermo Fisher Scientific, Waltham, MA, United States), and 1% agarose gel was used to check the DNA quality. The V4 region of bacterial 16S rRNA gene was amplified with the universal primer set of 515F and 806R (Walters et al., 2016). Polymerase chain reactions (PCRs) contained 25 μL 2× Premix Taq (Takara, Dalian, China), 1 μL each primer (10 mM) and 3 μL DNA (20 ng/μL) template in a volume of 50 μL. Target DNA fragments were amplified by thermocycling: 5 min at 94°C for initialization; 30 cycles of 30-s denaturation at 94°C, 30-s annealing at 52°C, and 30-s extension at 72°C, followed by 10-min final elongation at 72°C. PCR products were purified with EZNA Gel Extraction Kit (Omega Bio-tek, Norcross, GA, United States). Sequencing libraries were generated using NEBNext Ultra DNA Library Prep Kit for Illumina^®^ (New England Biolabs, Ipswich, MA, United States) following the manufacturer’s protocols. The acquired libraries were assessed by Qubit 2.0 Fluorometer (Thermo Fisher Scientific, Waltham, MA, United States) and then sequenced on an Illumina HiSeq 2,500 platform with 250-bp paired-end reads (Guangdong Magigene Biotechnology Co., Guangzhou, China).

The abundance of bacteria in each sample was determined by quantitative PCR (qPCR) with the primer set of 331F: 5′-TCCTACGGGAGGCAGCAGT-3′ and 797R: 5′-GGACT ACCAGGGTATCTAATCCTGTT-3′ (Nadkarni et al., 2002). The qPCR was run in a 20-μL reaction volume containing 10 μL of TB Green Premix Ex Taq II (Takara Bio, Kusatsu, Japan), 1 μL of each primer (20 μM), 6 μL RNase-free water, 1 μL bovine serum albumin, and 1 μL template by CFX96Touch (Bio-Rad, Hercules, CA, United States). Each qPCR run was conducted with the following procedures: 95°C for 30 s, 40 cycles of 95°C for 1 s, 62°C for 30 s, and 72°C for 1 min (Xiang et al., 2014). Standard curves were constructed with 10-fold serially diluted linear pClone007 plasmids (Tsingke, Wuhan, China) containing targeted fragment from *Escherichia coli* DH5α (Takara Bio) for bacteria. The amplification efficiency ranged from 90 to 110% and the linear fitting showed a *R*^2^ > 0.99.

### Biolog EcoMicroplate Data Analysis

Utilization of organic carbon substrates in each well of Biolog EcoMicroplate was indicated by the chroma change due to the reduction of tetrazolium dye. The activity of microbial carbon utilization was measured by average well-color development (AWCD) (Garland and Mills, 1991). The functional diversities of microbial communities were determined as described previously (Keylock, 2005). The AWCD was calculated according to the following equations:

(1) $$\mathrm{AWCD}=\sum_{i=1}^{n}\left(C_{i}-R\right)/n$$

In eq. (1), *C*_*i*_ is the absorbance value of an individual reaction well, which is the deference value of between 590 and 750 nm; *R* is the absorbance value of control well; and *n* is the number of wells. If the value of (*C*_*i*_ − *R*) is negative, the absorbance is denoted as 0. The equations for calculating the McIntosh index (U) and Shannon index (*H*′) are as follows:

(2) $$U=\sqrt{\sum n_{i}^{2}}$$

(3) $$H^{\prime }=-\sum P_{i}\text{In}P_{i}$$

(4) $$P_{i}=\left(C_{i}-R\right)/\sum \left(C_{i}-R\right)$$

In equation (2), *n*_*i*_ is (*C*_*i*_ - *R*) of equation (1). In equations (3) and (4), *P*_*i*_ represents the ratio of the absorbance value of a specific well (1–31) to the total absorbance values of all wells. These indices reflected substrate utilization and functional diversity of microbial communities.

Each Biolog EcoMicroplate contains 31 carbon sources affiliated with six groups including carbohydrates, amino acids, amines, esters, carboxylic acids, and alcohols. For principal component analysis (PCA), redox signal intensities (*R*_*si*_) were normalized with AWCD using eq. (5) (Classen et al., 2003):

(5) $$R_{\text{si}}=\left(C_{i}-R\right)/\text{AWCD}$$

In this study, optical density at 72-h incubation was used for the calculation and statistical analysis (Garland, 1996).

### 16S rRNA Sequence Analysis

Paired-end raw sequences were performed according to Trimmomatic (v0.33,^1^) (Bolger et al., 2014) and assigned to the corresponding samples according to their barcode sequences. Sequences with ambiguous base “N,” quality score <20, and length <100 bp were discarded. High-quality sequences were subsequently merged using FLASH (v1.2.11,^2^) (Magoè and Salzberg, 2011) according to the overlap >10 bp between R1 and R2 reads and error ratio of the overlap region <0.1. Clean sequences were clustered at a 97% cutoff for each operational taxonomic unit (OTU) by USEARCH (v10,^3^) (Edgar, 2010), and singleton OTUs were filtered out. The sequences of mitochondria and chloroplast were removed, and the chimera sequences were identified by UCHIME algorithm (Edgar et al., 2011). The representative sequences for each OTU were categorized using Ribosomal Database Project classifier (v11.5,^4^) (Cole et al., 2014) and annotated referring to the SILVA (v132,^5^) (Pruesse et al., 2007). All samples were resampled to the same level (40,205 sequences) before further statistical analysis.

The original 16S rRNA gene data are available at the NCBI Sequence Read Archive^6^ with the accession number of PRJNA593490.

### Statistical Analysis

To investigate the impacts of WT and temperature on bacterial communities, nonparametric Kruskal–Wallis tests were conducted to discern the differences of physicochemical properties, alpha and functional diversities, and bacterial copies and taxonomy among samples from different months. Dunn test was used for multiple comparisons of them between bacterial communities collected in different months. The alpha diversity indices [Faith phylogenetic diversity (PD), observed species, Chao1 and Shannon] were conducted, and principal coordinate analysis (PCoA) of 16S rRNA sequences and PCA of carbon utilization data were conducted to visualize bacterial community dissimilarities across 3 months. The permutational multivariate analysis of variance (ADONIS) with the Bray–Curtis, Euclidean, and Jaccard metrics were used to test the structural and functional dissimilarities of bacterial communities across 3 months. The regressions between environmental factors and alpha and beta diversities were evaluated. The changes of community composition were performed by the chord diagram. Heatmaps revealed the carbon source preferences by bacterial communities and correlations between environmental factors, bacterial genus, and carbon sources. These analyses were performed with “vegan 2.5-7,” picante 1.8.2 “ggplot23.3.3,” “circlize 0.4.12,” “corrplot 0.84,” and “ggpubr 0.4.0” packages in R 3.5.1 (Oksanen et al., 2007; Kembel et al., 2010; Wickham, 2011; Gu et al., 2014; Wei, 2017; Kassambara, 2018). The relationships between bacterial communities, carbon sources, and environmental factors were performed with Monte-Carlo test of redundancy analysis (RDA) in Canoco 5.

The OTUs with relative abundances greater than 0.1% were selected across all samples, and the correlation matrices between OTUs and environmental factors were calculated. Spearman correlation coefficients (*r* > 0.7 or *r* < −0.7) with a significance of *P* < 0.01 [false discovery rate (FDR) corrected] were integrated into the network analysis. Each node represents one OTU or an environmental variable, and each edge represents a correlation between two nodes in the network. The co-occurrence network was characterized by topology indices including average degree, average path length, clustering coefficient, graph density, modularity, and network diameter. High values of average degree, clustering coefficient, and graph density suggest a more connected network, whereas higher average path length and diameter indicate loose associations in the network (Barberán et al., 2012). The node-level topological features (degree, betweenness, closeness, and eigenvector centrality) were calculated. High values of node topological features suggest the core position of a node in the network, whereas low values suggest a peripheral position (Ma et al., 2016). The real network was compared with 1,000 identical size Erdös-Réyni random networks (Erdös and Réyni, 1960). Nodes with high betweenness centrality values were considered as keystone species (González et al., 2010). These analyses were performed using “psych 2.0.12” and “igraph 1.2.6” packages in R (Csardi and Nepusz, 2006; Revelle, 2020), and network visualization was conducted with Gephi 0.9.2.

Indicator OTUs of microbial communities were determined using “indicspecies 1.7.9” package in R (De Cáceres and Legendre, 2009). In this study, all the OTU nodes of network were compared to determine indicator species in 3 months based on an indicator value >0.85 and *P* < 0.05 (FDR correction) after 999 permutation tests.

## Results

### Physicochemical Properties of Pore Water

All values of physicochemical parameters showed significant differences among 3 months except EC (*P* > 0.05) (Table 1). Values for DOC (10.25–22.88 mg/L and 11.28–17.38 mg/L), PWT (19.80–29.20°C and 19.90–26.90°C), and WT (−5.15 to 7.81 cm, −8.07 to 2.65 cm) were greater (*P* < 0.05) in May and August than in November (5.08–14.34 mg/L, 7.40°C –8.40°C, −14.04 to −1.06 cm, respectively), whereas values for DO (1.12–3.83 mg/L) and ORP (128.10–273.30 mV) were the greatest (*P* < 0.05) in November. All peat pore water samples were acidic (pH 3.98–5.98) and pH was significantly (*P* < 0.05) lower in May.

**TABLE 1:** Physicochemical properties of the peat pore water samples at the Dajiuhu peatland.

Month	DOC (mg/L)	DO (mg/L)	ORP (mV)	EC (μ S/cm)	pH	PWT (°C)	WT (cm)
May	15.23 ± 4.28^a^	0.34 ± 0.14^c^	84.77 ± 24.46^c^	16.67 ± 5.36^a^	4.53 ± 0.41^b^	22.78 ± 3.49^a^	1 ± 5^a^
August	14.24 ± 1.85^a^	1.34 ± 0.38^b^	147.63 ± 20.26^b^	18.14 ± 5.19^a^	5.18 ± 0.24^a^	23.55 ± 1.78^a^	−2 ± 4^a^
November	10.14 ± 3.58^b^	2.75 ± 0.89^a^	224.45 ± 41.19^a^	18.34 ± 3.80^a^	5.35 ± 0.24^a^	7.98 ± 0.36^b^	−7 ± 5^b^

*DOC, dissolved organic carbon; DO, dissolved oxygen; ORP, oxidation–reduction potential; EC, electrical conductivity; PWT, pore water temperature; WT, water table. WT is expressed with negative number when it is below the peat surface and *vice versa*. Values are means ± standard error (*n* = 12). Superscript letters within each column indicate significant difference (α = 0.05) based on Dunn multiple comparison tests. Same superscript indicates no significant difference between the parameters compared among different months, whereas different letters indicate significant difference among the comparison.*

### Diversities and Dissimilarities in Bacterial Communities

**FIGURE 1:**
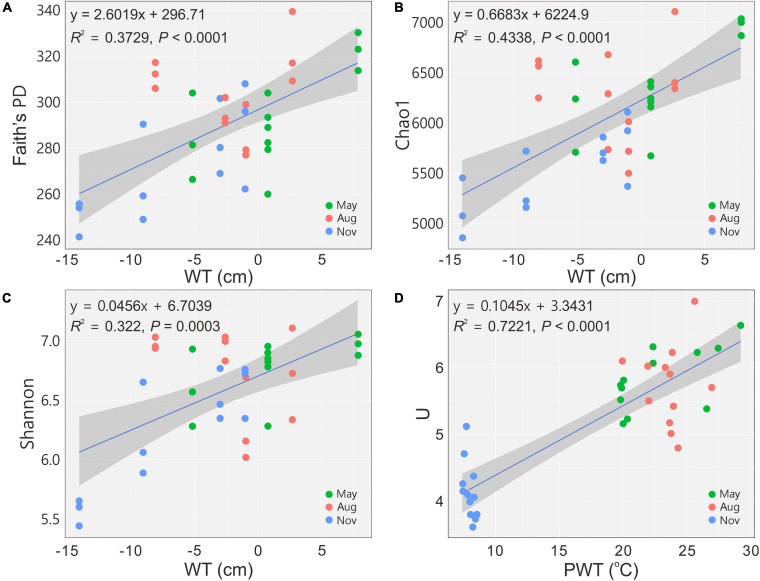
Changes in alpha diversity and functional diversity of bacterial communities with water table **(A–C)** and pore water temperature **(D)**, respectively. Blue solid lines indicate significant linear regressions (*P* ≤ 0.05, *n* = 36); gray background represents 95% confidence level. PD, phylogenetic diversity; U, McIntosh index; WT, water table; PWT, pore water temperature; Aug, August; Nov, November.

A total of 2,050,535 reads for 36 samples were obtained after quality control, and 12,380 OTUs were determined at a 97% similarity level. The indices of Faith’s PD, observed species, Chao1 and Shannon showed that the richness and diversity of bacterial communities were significantly (*P* < 0.05) higher in May and August (Table 2). The diversity indices *H*’ and *U* of carbon utilization by bacterial communities were also higher (*P* < 0.05) in May and August than those in November (Table 2). Bacterial 16S rRNA gene copies were significantly (*P* < 0.05) higher in May and August as indicated by qPCR (Table 2). The alpha diversity indices (Faith’s PD, Chao1 and Shannon) increased (regression model, *P* < 0.001) with the increase of WT (Figures 1A–C). Similarly, a significant positive relationship between U and PWT was observed (regression model, *P* < 0.0001) (Figure 1D).

**TABLE 2:** Structural and functional diversity indices and bacterial 16S rRNA gene copies of peat pore water samples collected in different months at the Dajiuhu peatland.

Month	16S rRNA gene sequencing			Biolog			16S rRNA quantification
	Faith’s PD	Observed OTUs	Chao1	Shannon	*U*	*H*′	lg (copies/mL peat water)
May	294 ± 22^a^	4263 ± 346^a^	6370 ± 448^a^	6.76 ± 0.26^a^	5.84 ± 0.47^a^	3.22 ± 0.07^a^	10.11 ± 0.21^a^
August	303 ± 17^a^	4334 ± 353^a^	6263 ± 463^a^	6.76 ± 0.39^a^	5.73 ± 0.61^a^	3.23 ± 0.12^a^	9.56 ± 0.24^b^
November	272 ± 22^b^	3707 ± 352^b^	5504 ± 380^b^	6.22 ± 0.48^b^	4.13 ± 0.44^b^	3.03 ± 0.16^b^	9.12 ± 0.31^c^

*PD, phylogenetic diversity; *U*, McIntosh index; *H*′, Shannon index. Values are means ± standard error (*n* = 12). Subscript letters within each column represent significant difference (α = 0.05) with Dunn multiple comparison tests. Same superscript indicates no significant difference between the parameters compared among different months, whereas different letters indicate significant difference among the comparison.*

**FIGURE 2:**
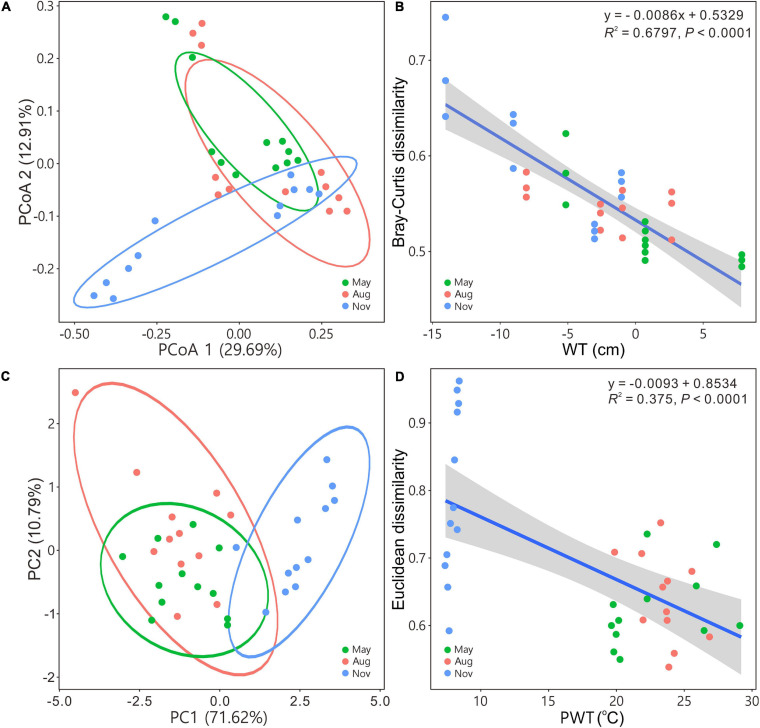
Principal coordinates analysis **(A)** and principal component analysis **(C)** illustrating bacterial community beta diversity among seasons. Ellipses represent 95% confidence interval. Variations in Bray–Curtis for bacterial communities and Euclidean dissimilarities for carbon metabolism with water table **(B)** and pore water temperature **(D)**, respectively. Blue solid lines indicate significant linear regressions (*P* ≤ 0.05, *n* = 36); gray background represents the 95% confidence level. The abbreviations are defined in Figure 1.

The PCoA and PCA indicated that bacterial communities in May and August were closely related to each other but separated from those in November (Figures 2A,C). Beta diversity based on Bray–Curtis, Euclidean, and Jaccard distances of bacterial assemblages were significantly different except those in May versus in August (Supplementary Table 1). Bray–Curtis and Euclidean dissimilarities significantly (regression models, *P* < 0.0001) decreased as WT (Figure 2B) and PWT increased (Figure 2D), respectively.

### Variations in Taxonomic Composition

**FIGURE 3:**
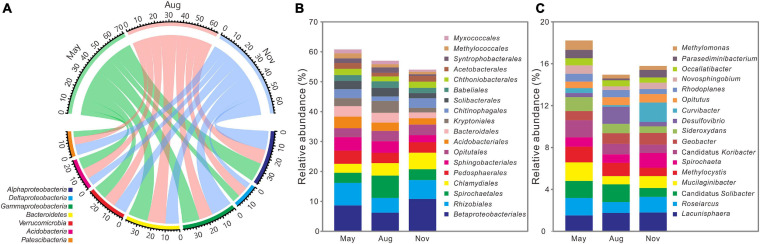
Distributions of the dominant phyla (relative abundance >5%) **(A)**, orders (relative abundance >1%) **(B)**, and genus (relative abundance >0.5%) **(C)** across 3 months (*n* = 36) at the Dajiuhu peatland. The numbers on the arc present the relative abundance.

All high-quality sequences were affiliated with 59 bacterial phyla and the main phyla (relative abundance >5%) were compared among 3 months (Figure 3A). Compared with bacterial communities in November, the relative abundances of *Deltaproteobacteria* (5.85 ± 1.00%, 7.88 ± 1.60%), *Bacteroidetes* (14.63 ± 3.33%, 13.67 ± 2.47%), and *Acidobacteria* (8.43 ± 2.03%, 7.90 ± 2.24%) were significantly (*P* < 0.05) higher in May and August (Supplementary Table 2), whereas that of *Gammaproteobacteria* (12.81 ± 2.76%, 10.32 ± 3.03%) were lower (*P* < 0.05). Among the main orders (relative abundance >1%), relative abundances of *Sphingobacteriales* (4.56 ± 1.53%, 3.88 ± 1.43%), *Acidobacteriales* (3.86 ± 1.32%, 2.79 ± 1.04%), *Bacteroidales* (3.54 ± 1.52%, 3.22 ± 1.66%), *Kryptoniales* (2.65 ± 1.54%, 3.94 ± 2.30%), *Solibacterales* (2.72 ± 0.85%, 2.89 ± 1.02%), *Syntrophobacterales* (1.56 ± 0.63%, 1.76 ± 0.71%), and *Myxococcales* (1.30 ± 0.38%, 1.19 ± 0.29%) were significantly (*P* < 0.05) higher in May and August, whereas *Betaproteobacteriales* (10.78 ± 5.27%), *Rhizobiales* (6.36 ± 2.29%), and *Chlamydiales* (5.53 ± 3.26%) dominated in November (Figure 3B and Supplementary Table 3). As for the main genus (relative abundance >0.5%), Candidatus *Solibacter* (1.62 ± 0.61%, 1.68 ± 0.64%) and *Methylocystis* (1.50 ± 0.52%, 1.17 ± 0.54%) were significantly (*P* < 0.05) enriched in May and August, whereas November had more *Spirochaeta* (1.72 ± 1.38%) (Figure 3C and Supplementary Table 4).

### Preference of Carbon Utilization

**FIGURE 4:**
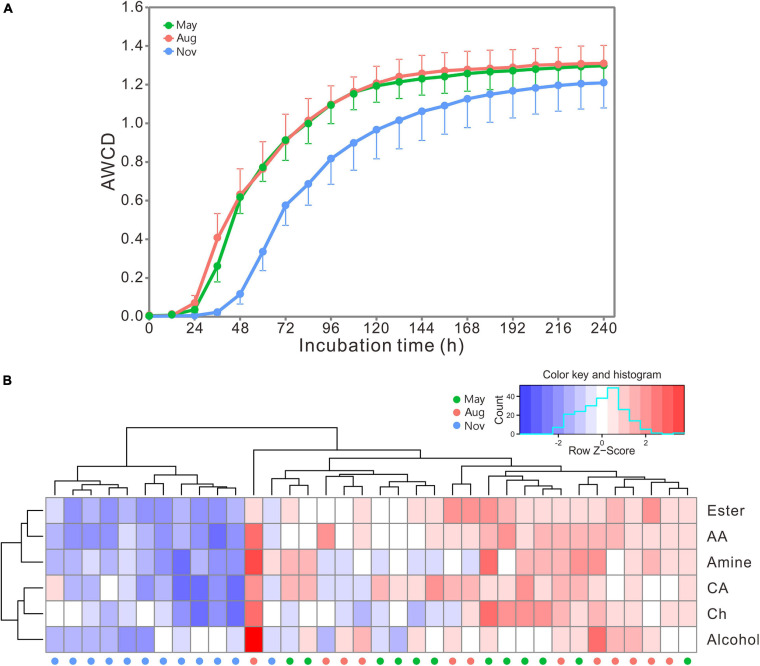
**(A)** The AWCD changes of carbon sources with incubation time. Bars represent the stand errors (*n* = 12). **(B)** Heatmap of bacterial preference for carbon source utilization. The color and histogram account for carbon metabolic rate of bacterial communities. AA, amino acid; CA, carboxylic acid; Ch, carbohydrate; Aug, August; Nov, November.

AWCD curves exhibited a lag phase in the first 24 h and increased dramatically from 24 to 72 h (Figure 4A). The increase slowed down afterward, and the carbon metabolic rate of bacterial communities stabilized beyond 168 h. The rate and preference of carbon utilization by bacterial communities were distinctly different among samples collected in different months as indicated by the heatmap (Figure 4B). Bacterial communities in May and August showed significantly higher metabolic rate of carbon utilization than those in November (Supplementary Table 5). However, all bacterial communities showed similar preference for carbon utilization. Overall, esters, amino acids and amines were preferentially utilized, followed by carbohydrates and carboxylic acids, and the alcohols were the lowest (Supplementary Table 6).

The loading scores of 31 carbon sources in the first two principal components are shown in Supplementary Table 7. The higher the loading scores were, the larger the effects of carbon sources in the principal components were. Overall, 10 carbon sources including four ester compounds (pyruvic acid methyl ester, Tween 40, Tween 80, and D-galactonic acid γ-lactone), two amino acids (L-arginine and L-threonine) and two amines (phenylethylamine and putrescine), one carboxylic acid substrate (itaconic acid), and one alcohol (D-mannitol) mainly impacted on the PC1. Besides putrescine, itaconic acid, Tween 40, and Tween 80, glucose-1-phosphate, glycyl-L-glutamic acid *N*-acetyl-D-glucosamine, and L-phenylalanine also impacted PC2 (Supplementary Table 7).

### Co-Occurrence Network

**FIGURE 5:**
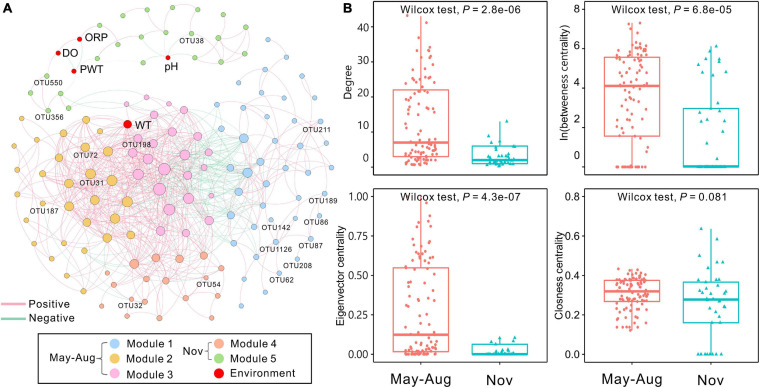
Co-occurrence network of bacterial communities among sampling months based on Spearman correlation analysis. **(A)** The nodes were colored by modularity. Each edge indicates a strong correlation (Spearman correlation coefficient of >0.7 or <–0.7) with a significance of *P* < 0.01 (false-discovery rate-corrected). The size of each node is proportional to the degree, and the thickness of edges is proportional to the absolute value of the Spearman correlation coefficient. Environmental variables and indicator OTUs are marked in the network. **(B)** Comparison of node-level topological features (degree, betweenness, eigenvector, and closeness centrality) of May–August and November bacterial communities. DO, dissolved oxygen; ORP, oxidation–reduction potential; PWT, pore water temperature; WT, water table. The significant differences between May–August and November are determined by Wilcoxon test (α = 0.05).

The bacterial network in peat pore water consisted of 142 nodes and 725 edges (Figure 5A). The results showed that more positive interactions were observed in peat pore water bacterial network, and the connections within a module were more intense compared with those among the modules. The degree of integrated network was distributed based on power law in peat pore water (Supplementary Figure 2A), indicating a scale-free distribution and nonrandom co-occurrence pattern compared with Erdös–Réyni random networks (Supplementary Figure 2B). The average path length was 3.62 with a diameter of 12. The average clustering coefficient was 0.556, and the modularity index was 0.547 (>0.4), which suggested a modular structure of the network (Newman, 2006). In the integrated network, WT had high values of degree, betweenness, and eigenvector centralities compared to other environmental factors (Figure 5A).

The 142 nodes were grouped into five modules in the network (Figure 5A). *Proteobacteria* were commonly found in all modules (Supplementary Table 8). Modules 1, 2, and 3 were dominated by *Proteobacteria* and *Bacteroidetes*. Nodes of module 4 were mainly belonged to *Patescibacteria*, *Proteobacteria*, and *Verrucomicrobia*. Module 5 was dominated by *Proteobacteria*. In total, 17 indicator OTUs were identified, and 12 of them were exclusively observed in May–August samples, and the other five indicator OTUs were found in samples of November (Supplementary Table 9). These obligate indicator OTUs were accordingly marked into the network and represented the distributions of bacterial communities in May–August and November. The indicator OTUs in May–August were in modules 1, 2, and 3, whereas those in November were affiliated with modules 4 and 5 (Figure 5A and Supplementary Table 9). Additionally, the node-level topological features (degree, betweenness centrality, and eigenvector centrality) significantly (*P* < 0.001) differed in May–August and November, whereas closeness centrality showed no significant differences (Figure 5B). According to betweenness centrality values, the top 10 nodes identified as keystone species were *Sphingomonas*, *Mucilaginibacter*, *Novosphingobium*, *Lacunisphaera*, *Herminiimonas*, and *Bradyrhizobium* (Supplementary Table 10).

### Relationship Among Bacterial Communities, Metabolic Functions, and Environmental Factors

**FIGURE 6:**
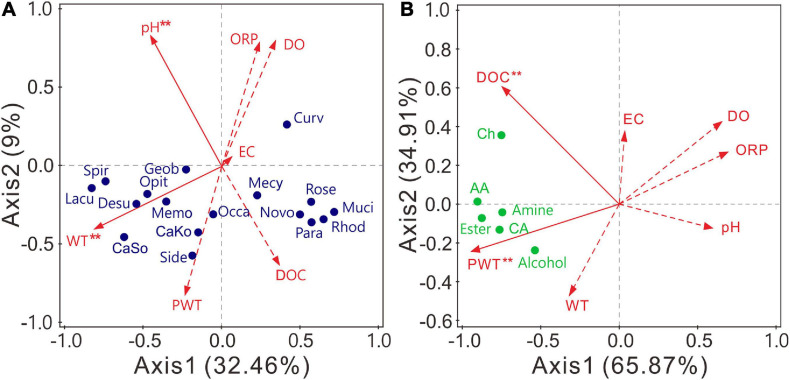
Redundancy analysis (RDA) indicating the influence of environmental variables on bacterial community composition **(A)** and carbon source utilization **(B)**. Variables with significant impacts (α = 0.05) are marked with red asterisk. Lacu, *Lacunisphaera*; Rose, *Roseiarcus*; CaSo, *Candidatus* Solibacter; Muci, *Mucilaginibacter*; Mecy, *Methylocystis*; Spir, *Spirochaeta*; CaKo, *Candidatus* Koribacter; Geob, *Geobacter*; Side, *Sideroxydans*; Desu, *Desulfovibrio*; Curv, *Curvibacter*; Opit, *Opitutus*; Rhod, *Rhodoplanes*; Novembero, *Novemberosphingobium*; Occa, *Occallatibacter*; Para, *Parasediminibacterium*; Memo, *Methylomonas*. DOC, dissolved organic carbon; DO, dissolved oxygen; ORP, oxidation–reduction potential; EC, electrical conductivity; PWT, pore water temperature; WT, water table. AA, amino acid; CA, carboxylic acid; Ch, carbohydrate.

RDA was applied to examine the effects of environmental factors on abundant bacterial genera (relative abundance >0.5%). The structures of bacterial communities were significantly (*P* < 0.05) affected by WT and pH (Figure 6A and Supplementary Table 11). Axis 1 and axis 2 explained 32.46 and 9% of the total variances, respectively. Candidatus *Koribacter*, *Candidatus* Solibacter, *Desulfovibrio*, *Geobacter*, *Lacunisphaera*, *Methylomonas*, *Opitutus*, *Sideroxydans*, and *Spirochaeta* were positively related to WT, whereas 10 of the 17 abundant genera negatively correlated with pH (Supplementary Figure 3A). In contrast, the carbon utilization of bacterial communities was significantly (*P* < 0.05) affected by PWT and DOC. Axis 1 and axis 2 explained 65.87 and 34.91% of the total variances, respectively (Figure 6B). The utilization of all carbon sources was positively related to the PWT and DOC (Supplementary Figure 3B). Altogether, the structure and carbon metabolism of bacterial communities were mainly shaped by WT and PWT, respectively.

## Discussion

### Different Impacts of WT and Temperature on Bacterial Communities

Bacterial communities in natural ecosystems are usually constrained by multiple factors. By combining 16S rRNA sequencing and Biolog techniques, we can demonstrate that WT and pH significantly shaped the compositions of bacterial communities (Figure 6A), whereas temperature and DOC largely impacted the rate of carbon utilization (Figure 6B). Statistically, carbon utilization was dissociated from bacterial community composition, possibly indicating that different taxa in bacterial communities had similar metabolic functions (Louca et al., 2018). Such functional redundancy could be unaffected by variation in taxa with the season of sampling.

Bacterial communities from peat pore water were distinctly diverse between May–August and November in the Dajiuhu peatland. The increase in the WT showed positive correlations with bacterial alpha diversity (Figures 1A–C) and decreased the bacterial community dissimilarities (Figure 2B) based on 16S rRNA gene sequencing. WT fluctuation due to precipitations in May–August and November can directly affect bacterial communities by changing physicochemical properties. For example, DO and ORP (Table 1) were negatively related to the WT (Supplementary Figure 4). WT fluctuation can change the depth of the oxic–anoxic interface (Tian et al., 2019), resulting in the subsequent shifts in electron donors and acceptors (Brune et al., 2000; Daffonchio et al., 2006). High WT facilitates anaerobic processes, and low WT accelerates the biodegradation of organic matters due to the penetration of oxygen and expansion of the oxic zone. Furthermore, WT also impacted dissimilar bacterial communities (Figures 2A,B) and composition (Figures 3A–C) in peat pore water.

Within the ambient environmental range, increasing temperature enhances microbial activities and promotes the decomposition of organic matter (Staley et al., 2015). Low temperature slows down metabolic activity and contributes to organic matter sequestration (Davidson and Janssens, 2006; Sanz-Lázaro et al., 2011). In our results, the decrease in peat PWT from May (22.78 ± 3.49°C) and August (23.55 ± 1.78°C) to November (7.98 ± 0.36°C) substantially decreased bacterial functional diversity (Figure 1D) and the carbon utilization rate (Figure 4A). The decrease in the temperature also shifted functional community structure (Figure metricconverterProductID2C2C) and increased the dissimilarities in metabolic structures of bacterial communities (Figure 2D). DOC was positively related with the PWT (Supplementary Figure 4). The relatively high concentration of DOC (Table 1) in peat pore water samples of May and August may contribute to enhanced heterotrophic respiration and metabolic rates as indicated by RDA between carbon utilization and environmental variables (Figure 6B).

Despite the differences in bacterial communities over the three seasons, the preferences for organic carbon substrates remained similar. Microbial communities in pore water preferred to utilize esters, amino acids, and amines (Supplementary Tables 6, 7), possibly derived from decaying plant debris, microbial biomass, and soil organic matter. The decomposition rate of plant debris particularly in *Sphagnum*-dominated peatland was extremely low due to the recalcitrance of high-molecular-weight organic compounds (e.g., polyphenols, cellulose, hemicellulose, and lignin derivatives) derived from *Sphagnum* under acidic conditions (Bragazza et al., 2007; Pankratov et al., 2011; Robroek et al., 2016). Soil-borne fungi have extracellular enzymes that can degrade recalcitrant aromatic and aliphatic compounds such as those in peatlands (Thormann, 2006; Juan-Ovejero et al., 2020). These microbes have peroxidases, laccases, and ligninases, which can produce intermediates that can subsequently serve as carbon sources for heterotrophic bacteria. Fungal biomass outweighs bacteria and upon decay and lysis may also support bacterial growth. The pore water DOC data suggest that it is mainly sourced from microbial metabolites or biodegradation of organic matter pool, with a mean concentration of 18.07 ± 10.60 mg/L. Fractions of this DOC has been identified as humic-like compounds (Wang R. et al., 2018).

### WT Shaping the Co-occurrence Patterns of Bacterial Communities

Co-occurrence network analyses can illustrate the microbial complexity and interrelationship among community members (Barberán et al., 2012). It is increasingly evident that microbial communities are structured and form complex interconnected networks via modularity (Ma et al., 2016; Jiao et al., 2019). The bacterial network of peat pore water showed a clear modular structure and was grouped into five modules (Figure 5A). Nodes in the modules can perform different functions (Newman, 2006) or prefer different habitats (Tian et al., 2020), as well as respond to environmental variables (de Vries et al., 2012). The WT was strongly linked to bacterial communities in the co-occurrence network (Figure 5A). The WT had remarkably high degrees of betweenness and eigenvector centralities compared with other environmental factors, suggesting a significant influence of WT on bacterial communities.

In the network analysis, samples from May and August were treated as one group due to high similarities based on physicochemical parameters, alpha and beta diversities, community dissimilarities, taxonomic compositions, and carbon utilizations. Distinct indicator species between May–August and November may reflect WT fluctuation. In May–August, 12 indicator OTUs were identified and mapped into modules 1, 2, and 3 (Supplementary Table 9). Among these indicator OTUs or species, *Rhodoplanes* spp. grow in subsurface anoxic environments (Lodha et al., 2015) and are capable of denitrification (Hiraishi and Ueda, 1994), as well as nitrogen fixation (Too et al., 2018). Members of *Desulfomonile*, *Smithella*, and *Syntrophobacter* affiliated with *Deltaproteobacteria* are anaerobic and mesophilic bacteria and can potentially degrade hydrocarbons and oxidize propionate in peatlands (Harmsen et al., 1998; Gray et al., 2011; Schmidt et al., 2016). *Candidatus* Solibacter spp. are capable of utilizing various carbon sources and reducing nitrate and nitrite under acidic, water-logged conditions (Kanokratana et al., 2011). These species may prefer high WT and perform specific functions under anoxic conditions. In contrast, the five obligate indicator OTUs of November were located in modules 4 and 5 (Supplementary Table 9). Members of *Reyranella* and *Aquitalea* can decompose polycyclic aromatics and cellulose and contribute to plant litter degradation under aerobic conditions (Woo et al., 2014; Lee et al., 2017). Many *Dechloromonas* spp. are capable of nitrogen fixation and their relative abundances increase in forest with a low WT (Leppänen et al., 2015). Therefore, functional properties of these indicator species may indicate environmental changes, for example, in the WT and oxygen concentration. Indeed, WT fluctuation (1 cm in May, -2 cm in August, -7 cm in November) and changes in DO concentration (0.34 mg/L in May, 1.34 mg/L in August, and 2.75 mg/L in November) in peat pore water coincided well with the ecological functions of indicator species.

Based on the betweenness centrality scores, *Sphingomonas*, *Mucilaginibacter*, *Novosphingobium*, *Lacunisphaera*, *Herminiimonas*, and *Bradyrhizobium* were identified as keystone species, which were mainly in module 1 (Supplementary Table 10). Acid-tolerant and oligotrophic *Sphingomonas* spp. participate in microbial communities under extreme environmental conditions (Ogita et al., 2006). Members of *Mucilaginibacter* isolated in ombrotrophic peatlands are able to utilize a broad range of biopolymers, particularly polysaccharides and proteins (Pankratov et al., 2007). Our results showed that *Mucilaginibacter* was positively related with carbohydrates and amino acids (Supplementary Table 12). The phenolic compounds from *Sphagnum*-derived litter could be degraded by several *Novosphingobium* spp. (DeAngelis et al., 2013). *Herminiimonas* affiliated with *Proteobacteria* are capable of degrading aromatic hydrocarbons (Kim et al., 2014). *Novosphingobium* and *Herminiimonas* can also utilize aromatics and were positively correlated to amines in the study (Supplementary Table 12). *Bradyrhizobium* spp. are common nitrogen-fixing soil bacteria (Bragina et al., 2013) and provide nitrogen for microbial communities in the peat pore water. These keystone species play an important role in nutrient cycle and sustain the bacterial co-occurrence network with broad niche and versatile or specific metabolic functions in peatland ecosystems. Keystone OTUs with higher betweenness centrality exhibited much tighter connections with the WT compared to other environmental variables (Figure 5A), which further verifies the large impact of WT on bacterial communities.

### Microbial Variations in Context of Global Climate Change

Precipitation and temperature are two fundamental factors in the global climate change. In the middle reach of the Yangtze River, high precipitation is usually coupled with high temperature due to the impact of East Asian monsoon. Thus, it is challenging to distinguish how an individual environmental variable impacts bacterial communities. Seasonal sampling strategy and combination of DNA sequencing and Biolog technique enabled us to investigate bacterial communities along gradients of temperature and WT at the level of DNA and organic carbon metabolism. We clearly distinguished the impacts of the WT and temperature on bacterial communities. WT significantly influenced the structures of bacterial community such as composition, relative abundance, and diversity. Carbon utilization rate and metabolic diversity were largely controlled by temperature. The variation in the WT fluctuation without extreme drought and flooding events can change bacterial community structure, but not the bacterial degradation of organic matter (i.e., preference of carbon sources and rate of utilization) in the Dajiuhu peatland. These data indicate that within the time span of this study, microbial functions may be more stable than microbial compositions. This may be attributed to metabolic function redundancy in natural ecosystems. However, the impacts of extreme precipitation events such as the once-in-a-century flood in summer of 2020 and the depth of the WT over a long term on microbial communities remain unknown and merit further investigation.

## Conclusion

Water table and temperature impacted bacterial pore water communities at the DNA and organic carbon metabolic levels, respectively, in the Dajiuhu peatland. WT level mainly impacted the structures of bacterial communities (alpha diversity, dissimilarity, relative abundance, and composition) and microbial interactions (co-occurrence pattern). Temperature largely shaped the functional diversity, functional community dissimilarity, and carbon utilization rate. Bacterial groups interacted particularly intensely during May–August with high WT and high temperature. Keystone species were identified that were mainly attributed to the decomposition of organic matter and nitrogen fixation in oligotrophic and acid peatlands. They sustained complex connections in the bacterial network of peat pore water. The combination of 16S rRNA sequencing and Biolog technique can differentiate the impacts of the WT and temperature on bacterial taxa and associated functions. This study substantiates our understanding of microbial community responses to environmental variables in peatland ecosystems. However, long-term series of sampling and *in situ*/*in vivo* detection of microbial activity are warranted to corroborate the outcomes of this study.

## Data Availability Statement

The datasets presented in this study can be found in online repositories. The names of the repository/repositories and accession number(s) can be found in the article/Supplementary Material.

## Author Contributions

WT did the experiments, data analysis, figures drawing, and manuscript drafting. XX conducted the field work and involved in writing. HW designed the study, provided the financial support and wrote up the manuscript. All authors contributed to the article and approved the submitted version.

## Conflict of Interest

The authors declare that the research was conducted in the absence of any commercial or financial relationships that could be construed as a potential conflict of interest.

## Footnotes

1 http://www.usadellab.org/cms/?page=trimmomatic

2 https://ccb.jhu.edu/software/FLASH/

3 http://www.drive5.com/usearch/

4 http://rdp.cme.msu.edu/

5 https://www.arb-silva.de/

6 https://www.ncbi.nlm.nih.gov/sra
