# Supplementary material for: Differential Impacts of Water Table and Temperature on Bacterial Communities in Pore Water From a Subalpine Peatland, Central China
Source: Front Microbiol. 2021 May 28;12:649981. doi: 10.3389/fmicb.2021.649981 (PMC8193233; doi:10.3389/fmicb.2021.649981)
Supplement: Supplementary file 1 [file Data_Sheet_1.pdf]

Differential impacts of water table level and temperature on bacterial communities in pore water from a subalpine peatland, Central China

Wen Tian[1,2], Xing Xiang[1,3], Hongmei Wang[1,2]*

[1] *State Key Laboratory of Biogeology and Environmental Geology, China University of Geosciences, Wuhan, 430074, PR China*
[2] *School of Environmental Studies, China University of Geosciences, Wuhan, 430074, PR China*
[3] *College of life science, Shangrao Normal University, Shangrao, 334001, PR China*

[*] Corresponding author
wanghmei04@163.com; hmwang@cug.edu.cn

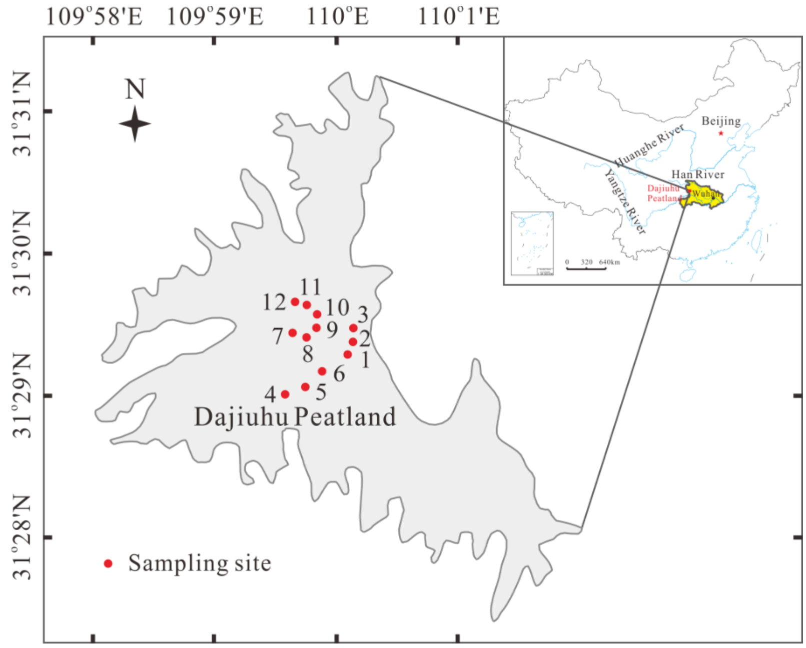

Supplementary Figure S1 | Location of sampling plot in this study

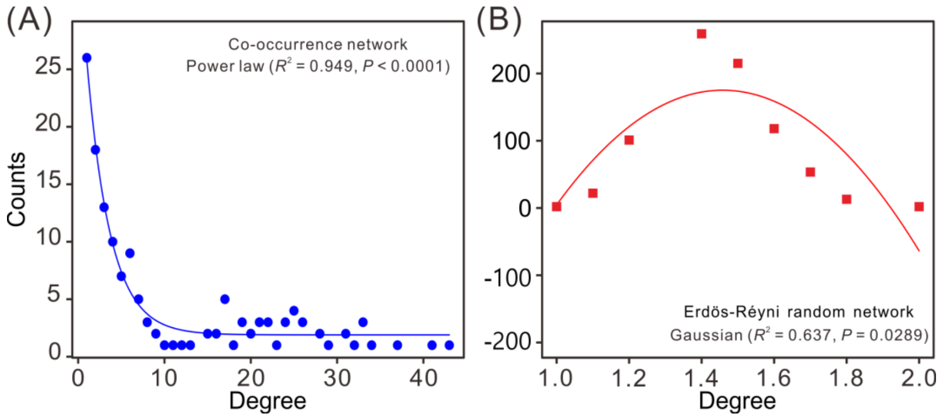

Supplementary Figure S2 | Distribution of degrees for the bacterial community co-occurrence network of pore water (a) and Erdös-Rényi random network (b) in the Dajiuhu Peatland.

(A)

	Lacu	Rose	CaSo	Muci	Mecy	Spir	CaKo	Geob	Side	Desu	Curv	Opit	Rhod	Novo	Occa	Para	Memo
DOC	-.505**	.454**	-.017	.393*	-.026	-.091	-.067	-.238	.317	.010	-.063	-.293	.375*	.452**	.197	.150	.072
DO	-.180	.072	-.426**	.011	-.133	-.148	-.280	-.149	-.501**	-.134	.437**	-.164	-.170	.104	-.335*	.109	-.214
ORP	-.070	.005	-.306	-.057	-.100	-.140	-.189	-.160	-.486**	-.091	.419*	-.107	-.208	.015	-.290	.037	-.205
EC	-.042	.255	-.009	.037	-.216	-.028	-.175	.230	-.082	.078	.247	.144	-.089	.198	.508**	.088	-.081
pH	.394*	-.455**	.056	-.518**	-.363*	.251	-.237	.049	-.485**	.230	.162	.114	-.523**	-.542**	-.251	-.248	-.273
PWT	-.081	-.054	.413*	.069	-.086	.221	.127	-.035	.431**	.315	-.368*	-.042	.258	-.050	.196	-.021	.092
WT	.541**	-.431**	.549**	-.475**	.029	.461**	.316	.206	.302	.300	-.527**	.230	-.335*	-.458**	.092	-.334*	.254

(B)

	Ch	AA	Ester	Alcohol	Amine	CA
DOC	.789**	.726**	.604**	.258	.542**	.493**
DO	-.332*	-.616**	-.632**	-.423*	-.499**	-.564**
ORP	-.420*	-.624**	-.664**	-.392*	-.548**	-.564**
EC	.109	-.022	.007	-.109	-.143	-.120
pH	-.483**	-.518**	-.537**	-.160	-.455**	-.510**
PWT	.630**	.864**	.871**	.586**	.735**	.735**
WT	.078	.321	.291	.334*	.234	.336

Supplementary Figure S3 | Correlation between environmental factors and bacterial genus (a), and carbon sources (b). Numeric values in panel represent the Pearson correlation coefficient. Lacu, *Lacunisphaera*; Rose, *Roseiarcus*; CaSo, Candidatus *Solibacter*; Muci, *Mucilaginibacter*; Mecy, *Methylocystis*; Spir, *Spirochaeta*; CaKo, Candidatus *Koribacter*; Geob, *Geobacter*; Side, *Sideroxydans*; Desu, *Desulfovibrio*;Curv, *Curvibacter*; Opit, *Opitutus*; Rhod, *Rhodoplanes*; Novo, *Novosphingobium*; Occa, *Occallatibacter*; Para, *Parasediminibacterium*; Memo, *Methylomonas*. DOC, dissolved organic carbon; DO, dissolved oxygen; ORP, oxidation-reduction potential; EC, electrical conductivity; PWT, pore water temperature; WT, water table. Ch, carbohydrate; AA, amino acid; CA, carboxylic acid. **, $P \leqslant 0.01$; *, $P \leqslant 0.05$.

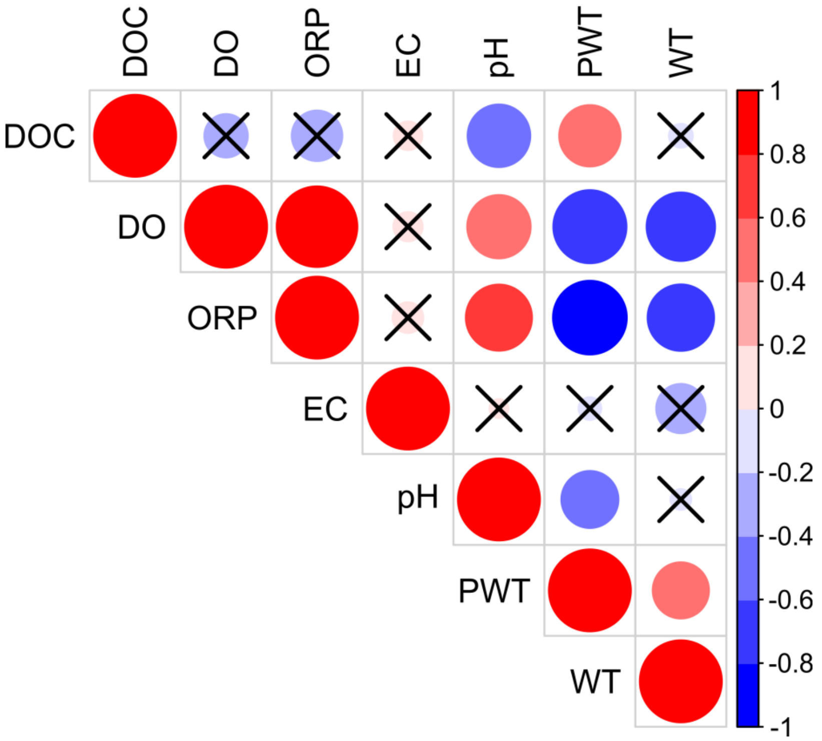

Supplementary Figure S4 | Correlation of environmental parameters. The heatmap depicts Pearson correlation where statistically significantly ($P < 0.05$) positive correlations (red), statistically significantly negative correlations (blue), and a lack of a significant correlation is marked by a cross.

Supplementary Table S1 | Dissimilarity test showing the differences in bacterial communities of pore water samples collected in May, August, and November at the Dajiuhu Peatland

Time factor	ADONIS							
	16S rRNA gene				Biolog			
	Bray-Curtis		Jaccard		Eulicdean		Jaccard	
	F	*P*	*R*	*P*	*R*	*P*	*R*	*P*
May *vs*. August	2.150	0.053	1.946	0.058	1.278	0.245	1.113	0.333
May *vs*. November	3.100	**0.006****	2.758	**0.008****	37.275	**0.001*****	31.420	**0.001*****
August *vs*. November	2.956	**0.008****	2.491	**0.011***	36.138	**0.001*****	31.060	**0.001*****
May *vs*. August *vs*. November	2.780	**0.002****	2.417	**0.001*****	23.606	**0.001*****	22.925	**0.001*****

ADONIS, permutational multivariate analysis of variance. Bold font represents significant value ($\alpha = 0.05$). Significance: ***, $P \leq 0.001$; **, $P \leq 0.01$; *, $P \leq 0.05$.

Supplementary Table S2 | Kruskal-Wallis test showing the differences in bacterial communities at the phylum/class level of pore water samples collected in May, August, and November at the Dajiuhu Peatland

Phylum/Class	May	August	November	Sig.
Alphaproteobacteria	14.19 ± 2.86%[a]	10.73 ± 4.27%[a]	14.26 ± 0.05%[a]	0.076
Deltaproteobacteria	5.85 ± 1.00%[b]	7.88 ± 1.60%[a]	4.16 ± 1.99%[c]	0.000
Gammaproteobacteria	12.81 ± 2.76%[b]	10.32 ± 3.03[b]	15.76 ± 6.17%[a]	0.012
Bacteroidetes	14.63 ± 3.33%[a]	13.67 ± 2.47%[a]	10.07 ± 5.98%[b]	0.028
Verrucomicrobia	10.21 ± 2.87%[a]	9.17 ± 1.66%[a]	9.77 ± 3.46%[a]	0.500
Acidobacteria	8.43 ± 2.03%[a]	7.90 ± 2.24%[a]	5.95 ± 2.83%[b]	0.040
Patescibacteria	4.52 ± 1.03%[a]	5.51 ± 1.71%[a]	8.08 ± 6.58%[a]	0.096

Values are means ± standard error (n = 12). Lowercase letters in the same row indicate significant difference (α = 0.05) based on Dunn's multiple comparison tests. Relative abundances decreasing from May-Aug to Nov are highlighted in red where those increase are in blue. Sig., significance.

Supplementary Table S3 | Kruskal-Wallis test showing the differences in bacterial communities at the order level of pore water samples collected in May, August, and November at the Dajiuhu Peatland

Order	May	August	November	Sig.
Betaproteobacteriales	8.65 ± 1.59%[ab]	6.23 ± 2.55%[b]	10.78 ± 5.27%[a]	0.012
Rhizobiales	7.53 ± 1.36%[a]	4.95 ± 2.52%[b]	6.36 ± 2.29%[ab]	0.019
Spirochaetales	3.37 ± 3.11%[a]	7.43 ± 6.84%[a]	3.62 ± 3.52%[a]	0.081
Chlamydiales	3.03 ± 0.85%[b]	4.20 ± 1.83%[ab]	5.53 ± 3.26%[a]	0.032
Pedosphaerales	4.37 ± 0.96%[a]	3.44 ± 0.73%[a]	3.47 ± 1.60%[a]	0.100
Sphingobacteriales	4.56 ± 1.53%[a]	3.88 ± 1.43%[a]	2.40 ± 1.35%[b]	0.003
Opitutales	2.95 ± 0.91%[a]	3.45 ± 1.52%[a]	3.52 ± 3.73%[a]	0.819
Acidobacteriales	3.86 ± 1.32%[a]	2.79 ± 1.04%[a]	2.11 ± 1.22%[b]	0.004
Bacteroidales	3.54 ± 1.52%[a]	3.22 ± 1.66%[a]	1.96 ± 1.60%[b]	0.050
Kryptoniales	2.65 ± 1.54%[a]	3.94 ± 2.30%[a]	1.54 ± 1.60%[b]	0.012
Chitinophagales	3.04 ± 3.24%[a]	1.52 ± 0.76%[a]	3.21 ± 4.58%[a]	0.387
Solibacterales	2.72 ± 0.85%[a]	2.89 ± 1.02%[a]	1.71 ± 1.61%[b]	0.047
Babeliales	1.96 ± 0.66%[a]	2.20 ± 0.56%[a]	1.73 ± 0.53%[a]	0.161
Chthoniobacterales	2.10 ± 2.39%[a]	1.61 ± 1.00%[a]	2.06 ± 1.59%[a]	0.749
Acetobacterales	1.90 ± 0.72%[a]	1.27 ± 0.63%[a]	1.92 ± 0.93%[a]	0.074
Syntrophobacterales	1.56 ± 0.63%[a]	1.76 ± 0.71%[a]	0.57 ± 0.24%[b]	0.000
Methylococcales	1.71 ± 1.42%[a]	1.04 ± 0.53%[a]	0.86 ± 0.81%[a]	0.104
Myxococcales	1.30 ± 0.38%[a]	1.19 ± 0.29%[a]	0.68 ± 0.40%[b]	0.000

Values are means ± standard error. Different lowercase letters in the same row represent significant difference at the 95% confidence interval with Dunn's multiple comparison tests. Relative abundances decreasing from May-Aug to Nov are highlighted in red where those increase are in blue. Sig., significance.

Supplementary Table S4 | Kruskal-Wallis test showing the differences in bacterial communities at the genus level of pore water samples collected in May, August, and November at the Dajiuhu Peatland

Genus	May	August	November	Sig.
Lacunisphaera	1.51 ± 0.74%[a]	1.75 ± 0.99%[a]	1.80 ± 0.02%[a]	0.857
Roseiarcus	1.69 ± 0.87%[a]	1.05 ± 0.56%[a]	1.49 ± 0.98%[a]	0.166
Candidatus *Solibacter*	1.62 ± 0.61%[a]	1.68 ± 0.64%[a]	0.85 ± 0.78%[b]	0.009
Mucilaginibacter	1.77 ± 1.27%[a]	0.80 ± 1.14%[a]	1.15 ± 1.37%[a]	0.174
Methylocystis	1.50 ± 0.52%a	1.17 ± 0.54%[a]	0.82 ± 0.43%[b]	0.008
Spirochaeta	0.89 ± 0.40%[b]	0.78 ± 0.59%[b]	1.72 ± 1.38%[a]	0.030
Candidatus *Koribacter*	1.63 ± 1.17%[a]	0.83 ± 0.30%[b]	0.79 ± 0.64%[bc]	0.022
Geobacter	0.86 ± 0.47%[a]	1.01 ± 0.50%[a]	1.11 ± 0.89%[a]	0.647
Sideroxydans	1.33 ± 0.39% [a]	0.90 ± 0.36% [b]	0.64 ± 0.48% [bc]	0.001
Desulfovibrio	0.40 ± 0.18%[bc]	1.63 ± 1.24%[a]	0.42 ± 0.46%[b]	0.000
Curvibacter	0.46 ± 0.46%[a]	0.14 ± 0.08%[a]	1.85 ± 3.23%[a]	0.077
Opitutus	0.62 ± 0.21%a	0.76 ± 0.30%[a]	0.81 ± 0.11%[a]	0.772
Rhodoplanes	0.74 ± 0.31%[a]	0.68 ± 0.67%[a]	0.48 ± 0.26%[a]	0.360
Novosphingobium	0.81 ± 0.38%[a]	0.36 ± 0.29%[a]	0.61 ± 0.59%[a]	0.051
Occallatibacter	0.68 ± 0.34%[a]	0.57 ± 0.36%[a]	0.50 ± 0.29%[a]	0.419
Parasediminibacterium	0.80 ± 1.97%[a]	0.17 ± 0.21%[a]	0.71 ± 2.22%[a]	0.631
Methylomonas	0.90 ± 1.09%[a]	0.36 ± 0.20%[a]	0.39 ± 0.54%[a]	0.127

Values are means ± standard error (n = 12). Lowercase letters in the same row indicate significant difference (α = 0.05) based on Dunn's multiple comparison tests. Relative abundances decreasing from May-Aug to Nov are highlighted in red where those increase are in blue. Sig., significance.

Supplementary Table S5 | Kruskal-Wallis test showing the differences of carbon utilization rate by bacterial communities of pore water samples collected in May, August, and November at the Dajiuhu Peatland

	May	August	November	Sig.
Esters	1.102 ± 0.125[a]	1.151 ± 0.152[a]	0.594 ± 0.103[b]	0.000
Amino acids	1.025 ± 0.123[a]	1.080 ± 0.121[a]	0.666 ± 0.084[b]	0.000
Amines	1.059 ± 0.188[a]	1.076 ± 0.197[a]	0.760 ± 0.136[b]	0.000
Carboxylic acids	0.914 ± 0.082[a]	0.821 ± 0.130[a]	0.514 ± 0.164[b]	0.000
Carbohydrates	0.781 ± 0.267[a]	0.770 ± 0.255[a]	0.522 ± 0.184[b]	0.002
Alcohols	0.599 ± 0.087[b]	0.728 ± 0.192[a]	0.473 ± 0.079[c]	0.000

Values are means ± standard error (n = 12). Different lowercase letters in the same row represent significant difference at the 95% confidence interval with Dunn's multiple comparison tests. Sig., significance.

Supplementary Table S6 | Kruskal-Wallis test showing the differences of carbon utilization preference by bacterial communities of pore water samples collected in May, August, and November at the Dajiuhu Peatland

	AWCD	Sig.
Esters	0.949 ± 0.284 [a]	0000
Amino acids	0.923 ± 0.215[a]	0000
Amines	0.965 ± 0.225[a]	0000
Carboxylic acids	0.750 ± 0.215[b]	0000
Carbohydrates	0.691 ± 0.261[b]	0000
Alcohols	0.600 ± 0.164[c]	0000

AWCD, average well-color development; Values are means ± standard error (n = 12). Different lowercase letters represent significant difference at the 95% confidence interval with Dunn's multiple comparison tests. Sig., significance.

Supplementary Table S7 | The 31 carbon sources loaded on the first and second principal component in analysis of Biolog EcoMicroplate data

Chemical guild	Plate number	Carbon source	PC1	PC2
Alcohols	C2	i-Erythritol	-0.065	0.021
	D2	D-Mannitol	0.193	-0.139
	H2	D,L-α-Glycerol phosphate	-0.065	-0.064
Amino acids	A4	L-Arginine	0.170	-0.105
	B4	L-Asparagine	-0.027	0.006
	C4	L-Phenylalanine	0.026	0.092
	D4	L-Serine	-0.001	-0.058
	E4	L-Threonine	0.145	-0.059
	F4	Glycyl-L-Glutamic acid	-0.051	0.092
Amines	E2	N-Acetyl-D-Glucosamine	-0.029	0.056
	G4	Phenylethylamine	0.054	0.032
	H4	Putrescine	0.065	0.093
Carbohydrates	E1	α-Cyclodextrin	-0.017	0.002
	F1	Glycogen	-0.094	-0.192
	G1	D-Cellobiose	-0.009	0.011
	H1	α-D-Lactose	-0.086	0.002
	A2	ß-Methyl-D-Glucoside	-0.095	-0.003
	B2	D-Xylose	-0.089	0.010
	G2	Glucose-1-Phosphate	-0.095	0.273
Carboxylic acids	F2	D-Glucosaminic acid	0.009	-0.024
	B3	D-Galacturonic acid	-0.025	0.019
	C3	2-Hydroxy benzoic acid	0.031	-0.110
	D3	4-Hydroxy benzoic acid	0.019	-0.149
	E3	γ-Hydroxybutyric acid	0.045	-0.062
	F3	Itaconic acid	0.132	0.181
	G3	α-Ketobutyric acid	-0.015	-0.082
	H3	D-Malic acid	0.032	0.008
Esters	B1	Pyruvic acid methyl ester	0.057	-0.149
	C1	Tween 40	0.124	0.076
	D1	Tween 80	0.127	0.051
	A3	D-Galactonic acid γ-Lactone	0.107	-0.015

Supplementary.**Table S8 |** The number of nodes for taxonomic compositions in each module in the bacterial co-occurrence network of pore water samples collected in May, August, and November at the Dajiuhu Peatland

Taxonomy	Module 1	Module 2	Module 3	Module 4	Module 5
Acidobacteria	3	9	0	1	0
Actinobacteria	3	0	0	0	0
Armatimonadetes	1	0	0	0	0
Bacteroidetes	8	6	7	0	4
Chloroflexi	0	1	2	0	0
Chlamydiae	0	0	0	0	5
Cyanobacteria	0	0	0	0	1
Dependentiae	0	1	0	0	0
Nitrospirae	0	1	0	0	0
Omnitrophicaeota	0	0	1	1	0
Patescibacteria	0	0	1	6	1
Planctomycetes	1	0	0	0	0
Proteobacteria	24	5	6	5	11
Rokubacteria	0	0	1	0	0
Spirochaetes	0	0	3	1	0
Verrucomicrobia	3	7	3	4	1

Supplementary Table S9 | Indicator OTUs for bacterial communities of pore water samples collected in May, August, and November at the Dajiuhu Peatland

OTU_ID	Modularity class	Indicator value	*P*-corrected	Relative abundance (%)	Taxonomy
**May-August (n = 12)**					
OTU_211	1	0.889	0.012*	0.12%	k_*Bacteria*; p_*Proteobacteria*; c_*Alphaproteobacteria*; o_*Rhizobiales*; f_*Xanthobacteraceae*; g_*Rhodoplanes*
OTU_142	1	0.859	< 0.001***	0.24%	k_*Bacteria*; p_*Proteobacteria*; c_*Deltaproteobacteria*; o_*Syntrophobacterales*; f_*Syntrophaceae*; g_*Desulfomonile*
OTU_187	2	0.883	< 0.001***	0.12%	k_*Bacteria*; p_*Proteobacteria*; c_Deltaproteobacteria; o_*Syntrophobacterales*; f_*Syntrophaceae*; g_*Smithella*
OTU_198	3	0.875	0.003**	0.10%	k_*Bacteria*; p_*Proteobacteria*; c_*Deltaproteobacteria*; o_*Syntrophobacterales*; f_*Syntrophobacteraceae*; g_*Syntrophobacter*
OTU_1126	1	0.932	0.006**	0.19%	k_*Bacteria*; p_*Proteobacteria*; c_*Gammaproteobacteria*; o_*Betaproteobacteriales*; f_*Burkholderiaceae*; g_*Herminiimonas*
OTU_189	1	0.944	0.007**	0.10%	k_*Bacteria*; p_*Proteobacteria*; c_*Gammaproteobacteria*; o_*Betaproteobacteriales*; f_*Burkholderiaceae*; g_*Duganella*
OTU_208	1	0.863	< 0.001***	0.40%	k_*Bacteria;* p_*Proteobacteria*; c_*Gammaproteobacteria*; o_*Betaproteobacteriales*; f_*Gallionellacea*e; g_*Sideroxydans*
OTU_86	1	0.935	< 0.001***	0.16%	k_*Bacteria*; p_*Proteobacteria*; c_*Gammaproteobacteria*; o_*Pseudomonadales*; f_*Pseudomonadaceae*; g_*Pseudomonas*
OTU_31	2	0.856	0.004**	0.37%	k_*Bacteria*; p_*Acidobacteria*; c_*Acidobacteriia*; o_*Solibacterales*; f_*Solibacteraceae_Subgroup_3*; g_Candidatus *Solibacter*
OTU_62	1	0.912	0.013*	0.15%	k_*Bacteria*; p_*Bacteroidetes*; c_*Bacteroidia*; o_*Bacteroidales*; f_*Paludibacteraceae*; g_*Paludibacter*
OTU_72	2	0.858	0.026*	0.13%	k_*Bacteria*; p_*Bacteroidetes*; c_*Ignavibacteria*; o_*Kryptoniales*; f_*BSV26*
OTU_87	1	0.9	< 0.001***	0.19%	k_*Bacteria*; p_*Bacteroidetes*; c_*Bacteroidia*; o_*Sphingobacteriales*; f_*env.OPS_17*
**November (n = 5)**					
OTU_54	4	0.977	0.040*	0.20%	k_*Bacteria*; p_*Proteobacteria;* c_*Alphaproteobacteria*; o_*Reyranellales*; f_*Reyranellaceae*; g_*Reyranella*
OTU_32	4	0.942	0.021*	0.38%	k_*Bacteria;* p_*Proteobacteria*; c_*Gammaproteobacteria*; o_*Betaproteobacteriales*; f_*Chromobacteriaceae*; g_*Aquitalea*

OTU_ID	Modularity class	Indicator value	*P*-corrected	Relative abundance (%)	Taxonomy
OTU_38	5	0.881	0.031*	0.33%	k_*Bacteria*; p_*Proteobacteria*; c_*Gammaproteobacteria*; o_*Betaproteobacteriales*; f_*Rhodocyclaceae*; g_*Dechloromonas*
OTU_356	5	0.853	0.008**	0.17%	k_*Bacteria*; p_*Chlamydiae*; c_*Chlamydiae*; o_*Chlamydiales*; f_*cvE6*
OTU_550	5	0.861	0.007**	0.11%	k_*Bacteria*; p_*Chlamydiae*; c_*Chlamydiae*; o_*Chlamydiales*; f_*cvE6*

P values are corrected by false discovery rate (fdr) in BH method. Significance: ***, $P \leq 0.001$; **, $P \leq 0.01$; *, $P \leq 0.5$.

Supplementary Table S10 | Keystone species in the bacterial co-occurrence network of pore water samples collected in May, August and November at the Dajiuhu Peatland

**OTU_ID**	**Group**	**Modularity class**	**Betweenness centrality**	**Taxonomy**
OTU_329	May-August	1	1,485	k_Bacteria; p_*Proteobacteria*; c_*Alphaproteobacteria*; o_*Sphingomonadales*; f_*Sphingomonadaceae*;g_*Sphingomonas*
OTU_17	May-August	1	1,413	k_Bacteria; p_*Bacteroidetes*; c_*Bacteroidia*; o_*Chitinophagales*; f_*Chitinophagaceae*
OTU_52	May-August	1	1,109	k_Bacteria; p_*Bacteroidetes*; c_*Bacteroidia*; o_*Sphingobacteriales*; f_*Sphingobacteriaceae*; g_*Mucilaginibacter*
OTU_6290	May-August	1	896	k_Bacteria; p_*Bacteroidetes*; c_*Bacteroidia*; o_*Sphingobacteriales*; f_*Sphingobacteriaceae*; g_*Mucilaginibacter*
OTU_42	May-August	1	554	k_Bacteria; p_*Proteobacteria*; c_*Alphaproteobacteria*; o_*Sphingomonadales*; f_*Sphingomonadaceae*; g_*Novosphingobium*
OTU_8122	May-August	3	521	k_Bacteria; p_*Verrucomicrobia*; c_*Verrucomicrobiae*; o_*Opitutales*; f_*Opitutaceae*; g_*Lacunisphaera*
OTU_2396	May-August	3	485	k_Bacteria; p_*Bacteroidetes*; c_*Bacteroidia*; o_*Bacteroidales*; f_*Bacteroidetes_vadinHA17*
OTU_1126	May-August	1	467	k_Bacteria; p_*Proteobacteria*; c_*Gammaproteobacteria*; o_*Betaproteobacteriales*; f_*Burkholderiaceae*; g_*Herminiimonas*
OTU_11236	November	4	454	k_Bacteria; p_*Verrucomicrobia*; c_*Verrucomicrobiae*; o_*Opitutales*; f_*Opitutaceae*
OTU_30	May-August	1	440	k_Bacteria; p_*Proteobacteria*; c_*Alphaproteobacteria*; o_*Rhizobiales*; f_*Xanthobacteraceae*; g_*Bradyrhizobium*

Top ten nodes with betweenness centrality values are identified as keystone species in co-occurrence networks.

Supplementary Table S11 | Environmental factors correlated to bacterial communities and carbon source metabolism in pore water samples corrected in May, August, and November at the Dajiuhu Peatland

Factor	Bacterial communities			Carbon metabolism		
	explained variation	*F*	*P*-corrected	explained variation	*F*	*P*-corrected
WT	22.9	10.1	**0.004****	1.2	1.1	0.466
DO	1.8	1.0	0.473	1.5	1.5	0.434
ORP	2.4	1.3	0.294	1.3	1.3	0.443
pH	14.4	7.6	**0.004****	0.7	0.7	0.636
PWT	3.6	2.0	0.133	58.6	48.1	**0.007****
DOC	0.4	0.2	0.978	7.4	7.2	**0.007****
EC	3.3	1.8	0.156	0.7	0.7	0.636
Total	36	--	--	64.2	--	--

WT, water table; DO, dissolved oxygen; ORP, oxidation-reduction potential; PWT, pore water temperature; DOC, dissolved organic carbon; EC, electrical conductivity; --, not applied.

P values are corrected by false discovery rate (fdr) in BH method. Bold font represents significant value ($\alpha = 0.05$).

Significance: **, $P \leq 0.01$; *, $P \leq 0.5$.

Supplementary Table S12 | Carbon source correlated to keystone species in pore water samples corrected in May, August, and November at the Dajiuhu Peatland

	Sphingomonas	*Mucilaginibacter*	*Novosphingobium*	*Lacunisphaera*	*Herminiimonas*	*Bradyrhizobium*
Carbohydrates	0.131	0.191	0.319	-0.26	-0.053	0.091
Amino acids	0.078	0.107	0.138	0.065	0.265	0.12
Esters	0.167	0.129	0.182	0.046	0.385*	0.017
Alcohols	-0.087	-0.142	-0.154	0.159	0.192	-0.246
Amines	0.26	0.251	0.338*	-0.251	0.337*	0.029
Carboxylic acids	0.192	0.201	0.187	-0.022	0.506**	-0.004

Values represent the Spearman correlation coefficient. Significance: **, $P \leq 0.01$; *, $P \leq 0.5$ ($\alpha = 0.05$).
